# Performance of a prediction method for activities of daily living scores using influence coefficients in patients with stroke

**DOI:** 10.3389/fneur.2024.1419405

**Published:** 2024-08-19

**Authors:** Ryu Kobayashi, Norikazu Kobayashi

**Affiliations:** ^1^Department of Occupational Therapy, School of Health Science, International University of Health and Welfare, Narita, Japan; ^2^Department of Occupational Therapy, Graduate School of Human Health Science, Tokyo Metropolitan University, Arakawa, Japan

**Keywords:** stroke, activities of daily living, functional independence measure, prediction, predictive performance, influence coefficient

## Abstract

**Introduction:**

Recently, a method was developed to predict the motor Functional Independence Measure (FIM) score at discharge in patients with stroke by stratifying the effects of factors such as age and cognitive function and multiplying those by the influence coefficients of these factors. However, an evaluation of the predictive performance of the method is required for clinical application. The present study aimed to evaluate the predictive performance of this prediction method.

**Methods:**

Patients with stroke discharged from a rehabilitation ward between April 2021 and September 2022 were included. Predicted values of the motor FIM score at discharge were calculated after data collection from the hospital’s patient database. The concordance between predicted and actual values was evaluated using the interclass correlation coefficient; moreover, the residual values were calculated.

**Results:**

In total, 207 patients were included in the analysis. The median age was 79 (69–85) years, and 112 (54.1%) patients were male. The interclass correlation coefficient between predicted and actual values was 0.84 (95% confidence interval 0.75–0.89) for the motor FIM score at discharge. Meanwhile, the median residual value was 5.3 (−2.0–10.3) for the motor FIM score at discharge.

**Discussion:**

The prediction method was validated with good performance. However, the residual values indicated that some cases deviated from the prediction. In future studies, it will be necessary to improve the predictive performance of the method by clarifying the characteristics of cases that deviate from the prediction.

## 1 Introduction

Stroke is a leading cause of disability. Long-term disabilities following a stroke may cause significant physical, mental, and financial burdens for patients and their families (1); moreover, stroke is a major health problem worldwide. In Japan, more than 1.1 million people suffer from stroke each year (2), and it is the second leading cause (only behind dementia) of long-term care requirement. Promoting independent living among patients with stroke remains an important social issue. Stroke rehabilitation is believed to play an important role in solving this social problem by promoting patients’ independent living.

Prognostic prediction is important for efficient and effective stroke rehabilitation (3). Accurate prognostic prediction is essential for setting attainable goals, providing information to patients and relatives, and making shared decisions (4). In particular, prognosis prediction of activities of daily living (ADL) has been of interest among clinicians and therapists because ADL dependency is associated with discharge destination and care burden.

Previous studies have attempted to predict the Functional Independence Measure (FIM) score, an ADL assessment scale, in patients with stroke using various methods (4–7). In Japan, several studies have used multiple regression analysis to predict the motor FIM (m-FIM) score at discharge, based on data such as the FIM score at admission and age, in patients with stroke in specialized rehabilitation wards (8–11). Multiple regression analysis is considered useful when there is a linear relationship between explanatory and objective variables. However, there is no linear relationship between the m-FIM score at admission or age and the m-FIM score at discharge (12). Therefore, the stratification of factors affecting the outcomes may be a useful method for a more accurate prediction of patient prognosis.

Recently, a novel prediction method that predicts the m-FIM score at discharge by stratifying the effects of factors such as age, cognitive function at admission, and duration from onset to admission and multiplying the influence coefficients of these factors was developed in Japan (12). This prediction equation consists of four variables routinely collected in rehabilitation wards in Japan, making it highly applicable in clinical practice. However, no study has examined the concordance between the predicted and actual values in this prediction method; in addition, its potential for clinical application has not been fully validated. The present study, therefore, aimed to evaluate the predictive performance of the prediction method in a validation cohort.

## 2 Materials and methods

### 2.1 Participants and setting

Patients with stroke diagnosed with cerebral infarction or hemorrhage and admitted to a specialized rehabilitation ward in a subacute care hospital between April 2021 and September 2022 were included in this retrospective study. The hospital is located in the northwestern part of Tokyo metropolitan area and has a total of 150 beds. All the patients with stroke underwent rehabilitation therapy every day during hospitalization. The rehabilitation programs included physical, occupational, and speech therapies, as necessary. Patients received a maximum of 3 h of rehabilitation therapy per day.

The exclusion criteria were the same as those in the development study (12): (1) patients with subarachnoid hemorrhage; (2) patients admitted within 7 days or > 60 days after onset; (3) patients who spent <14 days or > 180 days in the hospital; (4) patients transferred to acute care hospitals; (5) patients with medical conditions that worsened during hospitalization; and (6) patients with an m-FIM score of 91 points on admission.

This study was approved by the Ethics Committee of Tokyo Metropolitan University (approval no. 22066) and conducted in accordance with the “Transparent Reporting of a multivariable prediction model for Individual Prognosis Or Diagnosis” statement (13).

Owing to the retrospective design of the study, the participants were recruited on an opt-out method in lieu of informed consent. Data were accessed for research purposes from 20 December 2022 to 31 March 2023. During data collection, the first author had access to information that could identify individual participants.

### 2.2 Outcome measure

The FIM (14) was used to measure study outcomes. It is one of the most widely used tools for assessing ADL dependence in patients with disabilities and has proven to have a high reliability (15). The FIM consists of 18 items, including feeding, toileting, and communication. Each item is scored on a seven-point scale from 1 to 7, with higher scores indicating greater independence in ADL. Total FIM scores range from 18 to 126, m-FIM scores range from 13 to 91, and cognitive FIM (c-FIM) scores range from 5 to 35. The FIM scoring was done via a discussion between well-trained nurses and occupational therapists assigned to each patient. In the present study, m-FIM and c-FIM scores at admission and discharge were collected.

### 2.3 Variables

Demographic and clinical data necessary for prediction were collected from the hospital’s patient database. As in the development study (12), the following variables were investigated: age, sex, stroke type, duration from onset to admission (transfer interval), and length of hospital stay.

### 2.4 Prediction method

**Figure 1:**
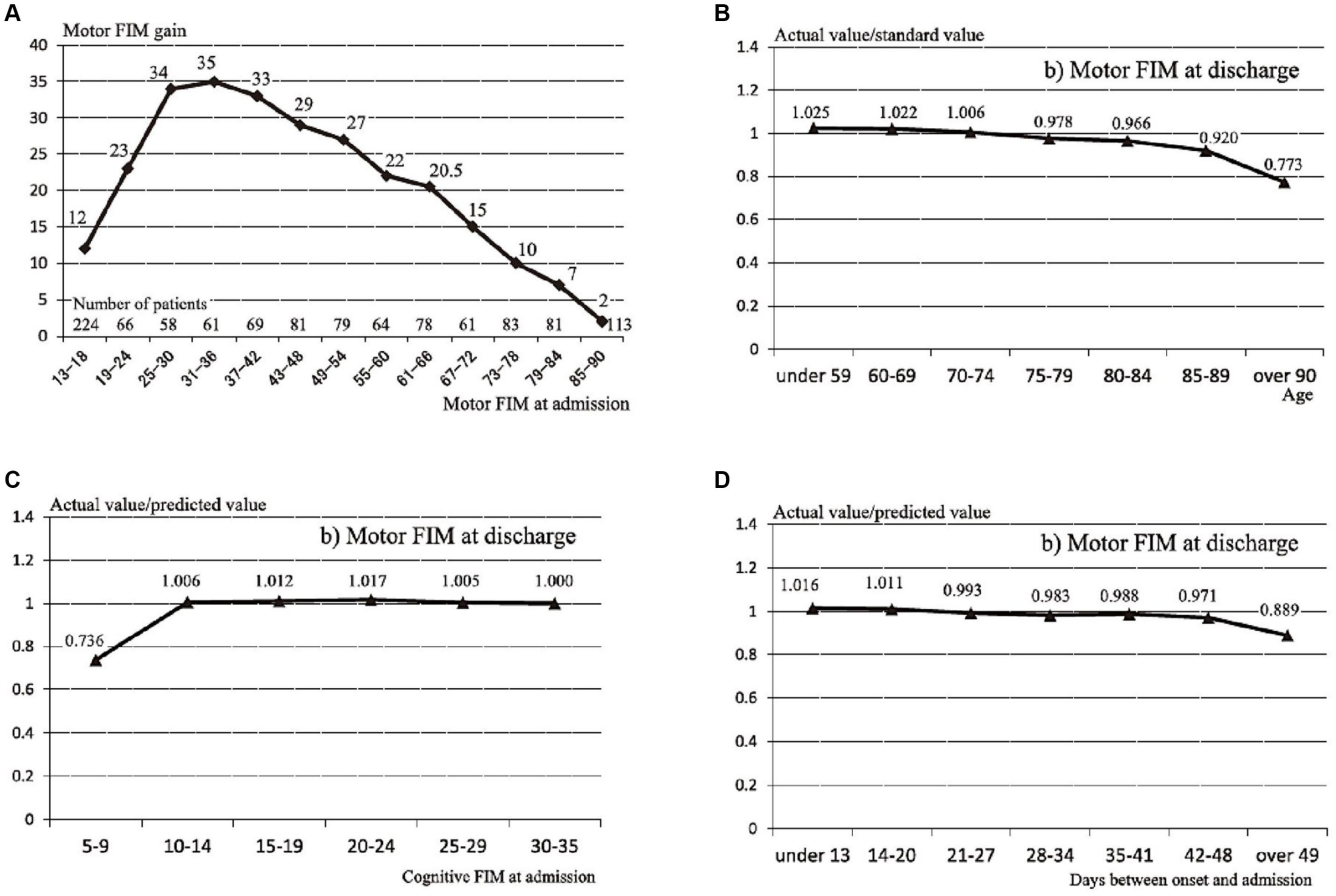
Standard values of motor Functional Independence Measure (FIM) gain and the influence coefficients for age, cognitive function, and transfer interval. **(A)** Standard values of motor FIM gain in 13 groups stratified by motor FIM score at admission. **(B)** Influence coefficient for age. **(C)** Influence coefficient for cognitive function. **(D)** Influence coefficient for transfer interval Reprinted from Tokunaga et al. (12).

The prediction method was as previously described (12). The first step in the prediction method is to calculate the standard value of the m-FIM gain from the m-FIM score at admission based on Figure 1A. The standard value of the m-FIM gain was the median m-FIM gain calculated for each group after classification of the participants in the previous study into 13 groups with the m-FIM score at admission in six-point increments. The standard value for m-FIM gain was calculated as 35 if the patient’s m-FIM score at admission was 32. The next step was to calculate each effect coefficient from age, cognitive function, and transfer interval according to Figures 1B–D. Finally, the predictive value of the m-FIM score at discharge was calculated using the following formula:


$$\begin{array}{l} \mathrm{The predictive value}\;\\ \mathrm{of the}\;m-\\ FIM\;\mathrm{score}\;\mathrm{at}\;\mathrm{discharge}=\left(\begin{array}{l} \mathrm{the standard value of the}\;m-\\ FIM\;\mathrm{gain}+\mathrm{the}\;m-\\ FIM\;\mathrm{score}\;\mathrm{at}\;\mathrm{admission} \end{array}\right)\times \\ \left(\begin{array}{l} \mathrm{the influence coefficient}\;\\ for\;\mathrm{age} \end{array}\right)\times \\ \left(\begin{array}{l} \mathrm{the influence coefficient}\;\\ \mathrm{for cognitive function} \end{array}\right)\times \\ \left(\begin{array}{l} \mathrm{the influence coefficient}\;\\ \mathrm{for transfer interval} \end{array}\right) \end{array}$$

For example, if the m-FIM score at admission was 28, age was 87 years, c-FIM score at admission was 12, and number of days from onset to admission was 40, the predictive value of the m-FIM score at discharge would be (28 + 34) × 0.920 × 1.006 × 0.988 = 56.7.

### 2.5 Statistical analyses

Statistical analyses were performed using IBM SPSS Statistics for Windows, version 28.0. (IBM Corp., Armonk, N.Y., United States). Statistical significance was set at *p* < 0.05. Descriptive statistics were reported for the demographic data of the participants. Data are reported as means and standard deviation or medians with interquartile range for continuous variables, according to normality. Categorical variables are reported as percentages. In addition, one-sample Wilcoxon and chi-square tests were performed using the data from the previous study as comparison values and compared with the data from the present study; the one-sample test is a useful method for comparing representative values of samples from a previous study with the data in the present study.

After calculating the predicted values of m-FIM score at discharge, interclass correlation coefficients (ICC [2.1]) with 95% confidence intervals (CIs) were determined to reveal the concordance between predicted and actual values. The ICC is a valid measure for assessing the degree of concordance when the same participants are measured in different ways; it has been used in previous studies to assess the performance of prediction models for FIM scores (16, 17). The minimum sample size required to detect an ICC of 0.6 with an expected precision of 0.1 and a CI of 95% was 159 (18). In addition, the residual values were calculated by subtracting the predicted values from the actual values.

Subsequently, to clarify the characteristics of cases that deviated from the predicted values, the characteristics were compared among three groups: (1) a group within ±10 points of the residual m-FIM score at discharge, (2) a group with a residual > 10 points, and (3) a group with a residual <−10 point. In a previous study, an m-FIM score < 50 points was defined as complete assistance, 50–69 points as incomplete assistance, ≥70 points as self-care independence, and ≥ 80 points as walking independence (19). In other words, a patient’s level of ADL independence was considered to change approximately every 10 points. Therefore, in this study, a case was defined as deviating from the prediction if the error between the predicted and actual values was >10 points. Therefore, the group with residuals within ±10 points was defined as “the group within the predicted range.” In addition, the group with residuals > 10 points was defined as “the group above the prediction” and the group with residuals < −10 points was defined as “the group below the prediction.” The Kruskal–Wallis test was used to compare the three groups, followed by the Dunn’s test for multiple comparisons.

## 3 Results

### 3.1 Participants characteristics

**Figure 2:**
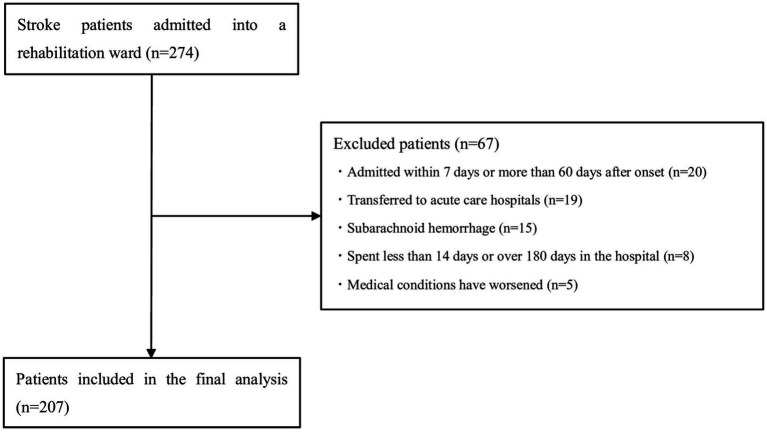
Study flowchart.

In total, 274 patients with stroke were admitted during the study period. Of these, 67 patients were excluded: 20 were admitted within 7 days or > 60 days after onset, 19 were transferred to acute care hospitals, 15 had subarachnoid hemorrhage, eight spent < 14 days or > 180 days in the hospital, and five had medical conditions that worsened during hospitalization. No patients had missing data. Therefore, 207 patients were finally included in the analysis (Figure 2).

Demographic variables are presented in Table 1. The median age was 79 (69–85) years, and 112 patients (54.1%) were male. One hundred and forty-six patients (70.5%) were diagnosed with cerebral infarction and 61 (29.5%) with hemorrhage. On admission, the median m-FIM score was 36 (20–53) and median c-FIM score was 18 (14–22); on discharge, the median m-FIM score was 73 (41–85) and median c-FIM score was 28 (18–33). In addition, the median m-FIM gain was 29 (14–34) and the median c-FIM gain was 7 (3–12).

**Table 1:** Comparison of demographic and clinical characteristics between the development and validation groups.

	Validation group	Development group (12)	*P*
	(*n* = 207)	(*n* = 1,118)	
Age, years (IQR)	79 (69–85)	72^†^	<0.001^a^
**Sex**			
Male, *n* (%)	112 (54.1)	680 (60.8)	<0.001^b^
Female, *n* (%)	95 (45.9)	438 (39.2)	
**Stroke type**			
Infarction, *n* (%)	146 (70.5)	716 (64.0)	<0.001^b^
Hemorrhage, *n* (%)	61 (29.5)	402 (36.0)	
Transfer Interval, days (IQR)	27 (21–36)	18^†^	<0.001^a^
Length of hospital stay, days (IQR)	116 (72–148)	81^†^	<0.001^a^
**FIM at admission**			
Motor score (IQR)	36 (20–53)	48.5^†^	<0.001^a^
Cognition score (IQR)	18 (14–22)	25^†^	<0.001^a^
**FIM at discharge**			
Motor score (IQR)	73 (41–85)	78^†^	<0.001^a^
Cognition score (IQR)	28 (18–33)	29^†^	<0.001^a^
**FIM gain**			
Motor FIM gain (IQR)	29 (14–34)	16^†^	<0.001^a^
Cognition FIM gain (IQR)	7 (3–12)	2^†^	<0.001^a^

FIM, Functional Independence Measure; IQR, interquartile range. Data were expressed as medians (IQR) and *n* (%). ^†^Interquartile range not stated. ^a^One-sample Wilcoxon test, ^b^one-sample Chi-Square test.

Compared to the development group, the validation group was significantly older, had a larger proportion of women, and had a longer transfer interval and length of stay (*p* < 0.001). Furthermore, the validation group had significantly lower m-FIM and c-FIM scores at admission and discharge but significantly higher FIM gains (*p* < 0.001).

### 3.2 Concordance and residuals between predicted and actual values in m-FIM scores at discharge

**Figure 3:**
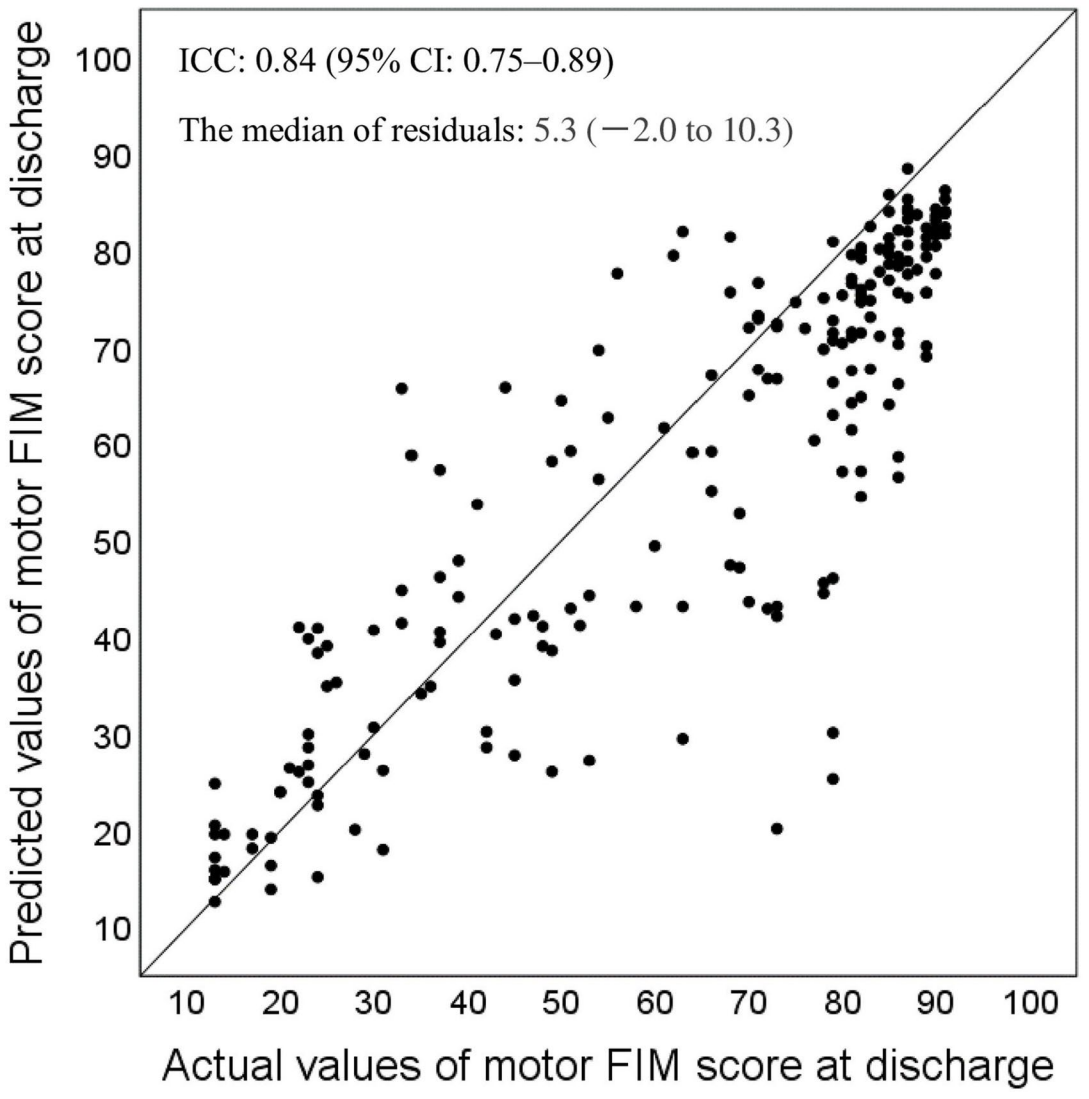
Relationship between the predicted and actual values for motor Functional Independence Measure (FIM) score at discharge, and the median residuals (*n* = 207). FIM, Functional Independence Measure; ICC, interclass correlation coefficient.

Figure 3 is a scatter plot showing the relationship between predicted and actual values in this prediction model. For the concordance between predicted and actual m-FIM scores at discharge, the ICC [2.1] was 0.84 (95% CI 0.75–0.89). Meanwhile, the median residual was 5.3 (−2.0–10.3).

### 3.3 Comparison of the three groups classified by residuals in the m-FIM scores at discharge

The three groups were classified according to residuals in the m-FIM scores at discharge; the results of the comparison of demographics and clinical data at admission are shown in Table 2. Only the m-FIM score at admission was significantly different between the three groups (*p* = 0.01). In multiple comparisons, the m-FIM scores at admission were significantly higher in the group within the predicted range than in the group above the prediction (*p* = 0.03). No significant differences were found between the other groups (*p* ≥ 0.05).

**Table 2:** Comparison of the three groups classified by the residuals in m-FIM scores at discharge.

Variables	(a) Groups within ±10 points of the residual	(b) Groups with residuals <−10 points	(c) Groups with residuals >10 points	P	Multiple comparison (*P*)
	(*n* = 136)	(*n* = 19)	(*n* = 52)		
Age, median (IQR)	78.0 (69.0–85.0)	80.0 (76.0–82.0)	79.0 (68.0–86.8)	0.89^†^	
**Sex**					
Male, *n* (%)	68 (50.0)	14 (73.7)	30 (57.7)	0.13^§^	
Female, *n* (%)	68 (50.0)	5 (26.3)	22 (42.3)		
**Stroke type**					
Infarction, *n* (%)	100 (73.5)	12 (63.2)	34 (65.4)	0.42^§^	
Hemorrhage, *n* (%)	36 (26.5)	7 (36.8)	18 (34.6)		
Duration from onset to admission, days, median (IQR)	27.0 (21.0–35.0)	26.0 (19.0–48.0)	30.0 (22.3–35.8)	0.68^†^	
**FIM at admission**					
Motor score, median (IQR)	45.5 (20.3–56.0)	27.0 (19.0–37.0)	27.5 (19.3–45.3)	0.01^†^	c < a (0.03)
Cognition score, median (IQR)	18.0 (14.0–22.0)	18.0 (13.0–21.0)	19.0 (16.3–21.0)	0.79^†^	

FIM, Functional Independence Measure; IQR, interquartile range. ^†^Kruskal–Wallis test; ^§^Chi-Square test.

## 4 Discussion

This study examined the predictive performance of a method for predicting the m-FIM scores at discharge using the standard values of FIM gain and influence coefficients for age, cognitive function, and transfer interval. The prediction model showed good predictive performance, as it exhibited high concordance between predicted and measured values.

Comparing the characteristics of the validation group in the present study with those of the development group in the previous study (12), there were significant differences in age, gender, transfer interval, length of hospital stay, stroke type, m-FIM scores at admission and discharge, and FIM gain. Despite these differences, the ICC [2.1] between the predicted and actual values was 0.84 (95% CI 0.75–0.89) in the validation group. Regarding ICC values, <0.50 indicate poor validity, 0.50–0.75 indicate moderate validity, 0.75–0.90 indicate good validity, and > 0.90 indicate excellent validity (20). These results indicate that the predictive performance of this prediction method is good.

Various methods have been reported to predict m-FIM scores at discharge in patients with stroke. Wada et al. tested the predictive accuracy in models using the reciprocal of m-FIM at admission and m-FIM effectiveness, and reported that the ICCs were 0.90 and 0.89 for each model (16). Kimura et al. (17) reported an ICC of 0.89 for their predictive model using a logarithmic model. However, using the above method would require collecting more data or data at two time points and complex calculations. The method investigated in this study has similar predictive accuracy to the method described above; however, our method is relatively simple, consisting of four variables that can be easily collected in a clinical setting. Therefore, this prediction method is superior in terms of ease of use in a clinical setting.

In contrast, the median residuals were 5.3 (−2.0–10.3) for the m-FIM score at discharge. The median residuals reported in the development study were 0 for the m-FIM score at discharge (12), and our results tended to show values higher than these. This suggests that some cases in the present study deviated from the predictions.

Therefore, to clarify the characteristics of the cases that deviated from the predictions at the time of admission, we compared the cases in three groups classified according to the residual in m-FIM scores at discharge. The results showed no significant differences in age, sex, stroke type, duration from onset to admission, and the c-FIM score at admission. Only the m-FIM score at admission showed a significant difference, being significantly higher in the group within the predicted range than in the group above the predicted range. However, there were no significant differences among the other groups. Therefore, it was difficult to clearly distinguish between groups that were within the predicted range and those that deviated from the predictions from the data collected at admission in this study.

Various factors have been reported to be associated with improved ADLs, including premorbid physical activity (21), lower severity of paresis (22), self-efficacy (23), improved nutrition during hospitalization (24), energy intake at admission (24), and interventions such as motor rehabilitation (3). Future studies on sensitivity and specificity are needed to identify patient populations with a greater predictive power, based on data collected from diverse perspectives.

This study had some limitations. First, it was performed at a single center in Japan; therefore, it is difficult to apply our results to cases from different regions and cultural backgrounds. The applicability of this predictive model in cases from different regions and cultural backgrounds needs to be verified in future studies. Second, only a few variables were employed in this prediction method. Future studies are needed to examine the impact of variables not included in the models, such as history of chronic disease and the amount and types of rehabilitation interventions, on the accuracy of outcome prediction; in addition, future studies attempting to update the models are required. Third, because this was a retrospective observational study, the limited variables collected at admission did not allow for adequate analysis of the groups for which this predictive tool would have high a predictive power. In the future, multidimensional data should be collected to clearly define the criteria for application of this tool.

This prediction method, as previously mentioned, has several challenges; however, the results of this study showed that the model demonstrated good predictive performance. The prediction method consists of four parameters that are easy to collect in routine clinical practice; therefore, we expect increased application of the prediction method in clinical practice in the future.

## 5 Conclusion

This study evaluated the prediction performance of a method to predict the m-FIM score at discharge by stratifying the effects of factors such as age, cognitive function at admission, and duration from onset to admission and then multiplying by influence coefficients of these factors. The results of this study showed high concordance between the predicted and actual values of the m-FIM scores at discharge. Meanwhile, the median residuals were 5.3 (−2.0–10.3) for the m-FIM score at discharge, suggesting that a certain number of patients deviated from the predictions. In future studies, clarifying the characteristics of cases that deviate from the predicted values is expected to further clarify the criteria for the application of this prediction method and improve its prediction accuracy.

## Data Availability

The data analyzed in this study is subject to the following licenses/restrictions: data are not authorized for release to the public by the Ethics Committee because they contain potentially personally identifiable patient information. Requests to access these datasets should be directed to the Ethics Committee of Tokyo Metropolitan University: a-rinri@jmj.tmu.ac.jp.
